# Superiority of a mechanistic codon substitution model even for protein sequences in Phylogenetic analysis

**DOI:** 10.1186/1471-2148-13-257

**Published:** 2013-11-21

**Authors:** Sanzo Miyazawa

**Affiliations:** 1 6-5-607 Miyanodai, Sakura, Chiba, 285-0857, Japan

**Keywords:** Amino acid substitution model, Empirical amino acid substitution rate matrix, Mechanistic codon substitution model, Structural constraints, Functional constraints, Selective constraints, Variable selective constraint across sites, Variable mutation rate across sites, multiple nucleotide change

## Abstract

**Background:**

Nucleotide and amino acid substitution tendencies are characteristic of each species, organelle, and protein family. Hence, various empirical amino acid substitution rate matrices have needed to be estimated for phylogenetic analysis: JTT, WAG, and LG for nuclear proteins, mtREV for mitochondrial proteins, cpREV10 and cpREV64 for chloroplast-encoded proteins, and FLU for influenza proteins. On the other hand, in a mechanistic codon substitution model, in which each codon substitution rate is proportional to the product of a codon mutation rate and the ratio of fixation depending on the type of amino acid replacement, mutation rates and the strength of selective constraint on amino acids can be tailored to each protein family with additional 11 parameters. As a result, in the evolutionary analysis of codon sequences it outperforms codon substitution models equivalent to empirical amino acid substitution matrices. Is it superior even for amino acid sequences, among which synonymous substitutions cannot be identified?

**Results:**

Nucleotide mutations are assumed to occur independently of codon positions but multiple nucleotide changes in infinitesimal time are allowed. Selective constraints on the respective types of amino acid replacements are tailored to each gene with a linear function of a given estimate of selective constraints, which were estimated by maximizing the likelihood of an empirical amino acid or codon substitution frequency matrix, each of JTT, WAG, LG, and KHG. It is shown that the mechanistic codon substitution model with the assumption of equal codon usage yields better values of Akaike and Bayesian information criteria for all three phylogenetic trees of mitochondrial, chloroplast, and influenza-A hemagglutinin proteins than the empirical amino acid substitution models with mtREV, cpREV64, and FLU, which were designed specifically for those protein families, respectively. The variation of selective constraint across sites fits the datasets significantly better than variable codon mutation rates, confirming that substitution rate variations across sites detected by amino acid substitution models are caused primarily by the variation of selective constraint against amino acid substitutions rather than the variation of codon mutation rate.

**Conclusions:**

The mechanistic codon substitution model is superior to amino acid substitution models even in the evolutionary analysis of protein sequences.

## Background

The reliability of phylogenetic analyses on protein-coding sequences strongly depends on models designed to approximate the substitution processes of nucleotide and amino acid. For the evolutionary analysis of protein-coding sequences, particularly phylogenetic inference, three types of substitution models can be used, provided that both DNA and protein sequences are available; nucleotide [1-3], amino acid [4-12], and codon substitution models [7,13-27]. For closely-related sequences in which most substitutions are synonymous, amino acid substitution models cannot be used, instead nucleotide substitution models may be employed. Conversely, nucleotide substitution models should not be used in the case of diverged sequences in which synonymous substitutions are almost saturated and nonsynonymous substitutions are significant. On the other hand, codon substitution models are appropriate to both closely-related and highly-diverged sequences with the intrinsic property of detecting both synonymous and nonsynonymous substitutions.

In a reversible Markov model for substitution, a substitution rate matrix must be specified to estimate the likelihood of a phylogenetic tree. Substitution tendencies between nucleotides and those between amino acids are characteristic of each species, each organelle, and each protein family. In the case of nucleotide substitution models, full parameterization for a substitution rate matrix is possible with 8 parameters; the total rate is normalized to one. However, 208 parameters for an amino acid substitution rate matrix are too many to be optimized for any size of a multiple sequence alignment. Thus, empirical amino acid substitution rate matrices have been estimated from a large number of substitutions inferred on phylogenetic trees of single or many protein families; the JTT [5], the WAG [10], and the LG [11] matrices from nuclear proteins, mtREV [6] from vertebrate mitochondrial proteins, cpREV10 [8] and cpREV64 [28] from chloroplast-encoded proteins, and FLU [29] from influenza proteins. Even a codon substitution rate matrix, KHG [25], has been estimated. These matrices significantly differ from one another, indicating that actually substitution tendencies significantly differ among these protein families. The estimation of a substitution rate matrix requires a large number or size of alignments with intensive calculation, and therefore is not always feasible. However, generic empirical substitution rate matrices such as JTT, WAG, LG,and KHG represent the average tendencies of substitutions over various protein families by sacrificing gene-level resolution [23]. On the other hand, a rate matrix such as mtREV, cpREV64, and FLU derived from a specific protein family represents substitution tendencies characteristic of the protein but often lacks generic representation of substitution tendencies enough to be applied to other protein families. To resolve this situation, the parametrization of an amino acid substitution rate matrix has been attempted to easily generate an alignment-specific rate matrix [30,31]. Here, we propose a different approach of employing a mechanistic codon substitution model in which the biological and evolutionary mechanisms of amino acid substitutions are taken into account.

In mechanistic codon substitution models [7,13-24,26,27], substitution rates are represented as the product of a codon mutation rate and the ratio of fixation, which depend on the types of codon replacement and amino acid replacement, respectively. Hence, mutational tendencies at the nucleotide level and selection at the amino acid level can be taken into account at various levels of separation. This is a critical difference from amino acid substitution models. As a result, the variations of codon mutation rate and selective constraint across sites can be distinguished from each other. We formulated a codon substitution rates between codons *μ* and *ν* as $R_{\mu \nu }\equiv M_{\mu \nu }(f_{\nu }/f_{\nu }^{\text{mut}})e^{w_{a_{\mu }b_{\nu }}}$, with a codon mutation rate *M*_*μν*_, a mutation equilibrium frequency $f_{\nu }^{\text{mut}}$, an equilibrium frequency *f*_*ν*_, and a selective constraint $w_{a_{\mu }b_{\nu }}$ on the substitutions between amino acids *a*_*μ*_ and *b*_*ν*_[26,27]. On the basis of this mechanistic codon substitution model, we estimated the *w*_*ab*_ by maximizing the likelihood of an empirical amino acid or codon substitution frequency matrix, each of JTT, WAG, LG, and KHG [26]. It was shown [27] that the mechanistic codon substitution model with a fully-parameterized codon mutation rate matrix (*M*) and a selective constraint matrix (*w*), each element of which was approximated as a linear function ( $w_{\text{ab}}=\min[\beta w_{\text{ab}}^{\text{estimate}}+w_{0}(1-\delta_{\text{ab}}),0]$) of a given estimate ( $w_{\text{ab}}^{\text{estimate}}$) of selective constraint estimated from the empirical substitution frequency matrix such as JTT and LG, outperformed both nucleotide and amino acid substitution models converted into codon substitution models in the wide range of codon sequences, from closely-related to highly-diverged protein-coding sequences. In this codon substitution model, codon mutations with multiple nucleotide changes are also taken into account, and were shown to increase significantly the likelihood of observed substitutions [26,27]. There have been a variety of models [7,13-24] for selective pressure on amino acid replacements in mechanistic codon substitution models. For their details, please see [27], in which the present mechanistic codon substitution model was discussed in comparison with other models.

In these days, DNA sequences are first analyzed and protein sequences are translated from them, and consequently codon sequences are likely available for most protein sequences in databases. However, there are protein sequences whose codon sequences are not available or not easily retrieved because of no cross link. In such a case, analysis at the amino acid level must be forced. Here we show that even for amino acid sequences, among which synonymous substitutions cannot be identified, the present mechanistic codon substitution model with the assumption of equal codon usage outperforms amino acid substitution models using empirical substitution rate matrices.

## Results and discussion

On the basis of Akaike Information Criterion (AIC) [32] and Bayesian Information Criterion (BIC) [33], the amino acid substitution models with the empirical amino acid substitution rate matrices, cpREV64 [28], cpREV10 [8], mtREV [6], and FLU [29], as well as JTT [5], WAG [10], and LG [11] that were estimated from nuclear proteins, are compared with the mechanistic codon substitution models [26,27] with the selective constraint matrices estimated from JTT, WAG, LG, and KHG [25] by using the 3 datasets: fast-evolving interspecific mitochondrial proteins (mammalian-mtProt) concatenating 12 protein-coding genes from 69 mammalian species [34], closely-related chloroplast-encoded proteins (cpProt-55) concatenating 52 protein-coding genes from 55 chloroplast genomes of the major angiosperm lineages [35], and HA proteins of Human influenza-A H1N1 (HA_Human-Flu-A-H1N1) consisting of 1309 sequences. The reference tree topologies used here as the most probable one are Tree-6 of [34] for mammalian-mtProt, the one reported in [35] for cpProt-55, and the one inferred by the FastTree version 2 [36] for HA_Human-Flu-A-H1N1. Branch optimization of phylogenetic trees and their maximum log-likelihood values are calculated using Phyml [37] modified for the mechanistic codon substitution model. Please see the Methods section for the details of the mechanistic codon substitution model and the protein sequence data used. The naming conventions of models employed here are described in Tables 1 and 2. The AIC and BIC values for these 3 datasets are listed in Tables 3, 4, and 5, respectively. Maximum log-likelihood (*ℓ*), AIC, and BIC values are represented in relative to those of the reference model, which uses the empirical amino acid substitution rate matrix specific to each dataset and has the lowest AIC and BIC values in all the amino acid substitution models; the best amino acid substitution model is chosen here as a reference model in order to show how superior mechanistic codon substitution models are in comparison with the best amino acid substitution model. A random effects approach (Bayesian mixture approach), in which the discrete gamma distribution [38,39] with *m* categories of unequal probabilities is assumed for a prior probability distribution, is employed for rate variation across sites in the amino acid substitution models, and also for the variation of selective constraint or mutation rate across sites in the mechanistic codon substitution models; see Additional file 1 for details. Suffixes "-dG*m*r" and "-dG*m*s" are appended to model names to denote rate and selective constraint variations across sites, respectively. The equilibrium frequencies of amino acids are assumed to be equal to those in the aligned sequences, and equal codon usage is assumed to calculate the equilibrium codon frequencies from them; a suffix "-F" is appended to the model names.

**Table 1:** Brief description of models: Amino acid substitution models

mtREV-dG*m*r, cpREV64-dG*m*r, FLU-dG*m*r,	The empirical amino acid rates of mtREV [6], cpREV64 [28], and FLU [29] are employed.
JTT-F-dG*m*r, WAG-F-dG*m*r, LG-F-dG*m*r,mtREV-F-dG*m*r, cpREV10-F-dG*m*r,cpREV64-F-dG*m*r, FLU-F-dG*m*r	The empirical amino acid exchangeabilities of JTT [5], WAG [10], LG [11], mtREV [6], cpREV10 [8], cpREV64 [28], and FLU [29] are employed. The suffix "-F" means that equilibrium amino acid frequencies are assumed to be equal to those of amino acid sequences.

The suffix "-dG*m*r" means that the variation of substitution rate is approximated by a discrete gamma distribution [38] with *m* categories of unequal probabilities; see Additional file 1 for details.

**Table 2:** Brief description of models: Mechanistic codon substitution models

Equal-Constraint-*n*-F-dG*m*(r|*s*|sf)	Equal constraint irrespective of amino acid substitution type is assumed; *β* = 0 in Eq. 4.
EI-*n*-F-dG*m*(r|*s*|sf)	$w_{\text{ab}}^{\text{EI}}\equiv -(\Delta {\hat{\varepsilon }}_{\text{ab}}^{c}+\Delta {\hat{\varepsilon }}_{\text{ab}}^{v}$) based on the Energy-Increment-based (EI) method [26] is used to estimate *w*_*ab*_ in Eq. 4. The $\Delta {\hat{\varepsilon }}_{\text{ab}}^{c}$ and $\Delta {\hat{\varepsilon }}_{\text{ab}}^{v}$ represent the effects of the mean increment of contact energy between residues and of residue-volume change due to an amino acid replacement, respectively; see Supporting Information, Text S1, in [26].
JTT-ML91+-*n*-F-dG*m*(r|*s*|sf), WAG-ML91+-*n*-F-dG*m*(r|*s*|sf), LG-ML91+-*n*-F-dG*m*(r |*s*|sf)	Selective constraints $\left\{w_{\text{ab}}^{\text{JTT/WAG/LG-ML91+}}\right\}$ estimated by maximizing the likelihood of JTT/WAG/LG [5,10,11] in the ML-91+ model [26] are used as $\left\{w_{\text{ab}}^{\text{estimate}}\right\}$ in Eq. 4.
KHG-ML200-*n*-F-dG*m*(r|*s*|sf)	Selective constraints $\left\{w_{\text{ab}}^{\text{KHG-ML200}}\right\}$ estimated by maximizing the likelihood of the KHG codon substitution matrix [25] in the ML-200 model [26] are used as $\left\{w_{\text{ab}}^{\text{estimate}}\right\}$ in Eq. 4.

The suffix "*n*" means the number of parameters optimized for the substitution rate matrix. The suffix "-F" means that equilibrium codon frequencies are assumed to be equal to codon frequencies in codon sequences; equal codon usage is assumed for amino acid sequences. The suffix "-dG*m*(r|*s*|sf)" denotes "-dG*m*r", "-dG*m*s" or "-dG*m*sf". The suffixes "-dG*m*r" and "-dG*m*s" mean the variation of mutation rate or selective constraint across sites, respectively, which is approximated by a discrete gamma distribution [38] with *m* categories of unequal probabilities; see Additional file 1 for details. The "f" following "-dG*m*s" means that the posterior frequencies of amino acids in each category in the first run are used in the second run as the equilibrium frequencies for each category; see the Methods section.

**Table 3:** Comparisons between various amino acid and codon substitution models for the reference phylogenetic tree of the mammalian-mtProt

**Substitution model**^**a**^	**K**^**b**^	**Δ*ℓ***^**c**^	**ΔAIC**^**c**^	**Δ**x**BIC**^**c**^	***β***^**de**^	***w***_***0***_^**d**^	${\langle e^{w_{\text{ab}}}\rangle }^{f}$	${\hat{m}}^{g}$	^**h**^	${\hat{\alpha }}^{i}$
Amino acid substitution models										
mtREV-dG4r	1	-96.5	154.9	37.2						0.471
cpREV64-F-dG4r	20	-3733.4	7466.9	7466.9						0.426
WAG-F-dG4r	20	-2667.4	5334.7	5334.7						0.443
LG-F-dG4r	20	-2617.5	5235.1	5235.1						0.438
cpREV10-F-dG4r	20	-2316.2	4632.4	4632.4						0.445
FLU-F-dG4r	20	-2249.4	4498.8	4498.8						0.433
JTT-F-dG4r	20	-1255.8	2511.6	2511.6						0.436
mtREV-F-dG4r	20	0.0	0.0	0.0						0.469
Mechanistic codon substitution models										
Equal-Constraint-10-F-dG4r	30	-3356.4	6732.7	6794.6	(0.0)	-0.000	1.000	0.338	2.887	0.407
EI-11-F-dG4r	31	-1663.4	3348.8	3417.0	0.463	0.012	0.276	0.369	4.061	0.424
WAG-ML91+-11-F-dG4r	31	356.4	-690.9	-622.8	1.140	0.017	0.122	0.336	3.978	0.427
LG-ML91+-11-F-dG4r	31	621.5	-1221.1	-1152.9	0.962	0.585	0.194	0.269	4.029	0.418
KHG-ML200-11-F-dG4r	31	701.5	-1380.9	-1312.8	1.321	0.944	0.223	0.196	1.939	0.415
JTT-ML91+-11-F-dG4r	31	712.6	-1403.2	-1335.1	1.354	0.539	0.137	0.348	2.417	0.421
JTT-ML91+-11-F-dG8r	31	1328.0	-2634.0	-2565.8	1.363	0.483	0.129	0.304	2.480	0.302
Equal-Constraint-10-F-dG4s	30	-3346.1	6712.1	6774.1	(0.0)	-0.000	1.000	0.300	2.950	0.396
EI-11-F-dG4s	31	-1164.7	2351.4	2419.5	0.553	-0.511	0.136	0.344	3.772	0.288
WAG-ML91+-11-F-dG4s	31	509.8	-997.6	-929.4	1.355	0.147	0.106	0.403	3.534	0.418
KHG-ML200-11-F-dG4s	31	511.1	-1000.2	-932.1	1.259	0.069	0.115	0.192	2.044	0.485
LG-ML91+-11-F-dG4s	31	637.6	-1253.2	-1185.1	0.994	-0.108	0.097	0.268	3.897	0.436
JTT-ML91+-11-F-dG4s	31	909.2	-1796.5	-1728.3	1.587	0.425	0.094	0.398	2.190	0.452
JTT-ML91+-11-F-dG8s	31	1712.7	-3403.4	-3335.2	1.739	0.409	0.078	0.348	2.250	0.328
Equal-Constraint-10-F-dG4sf	87	-1878.8	3891.7	4306.7	(0.0)	-0.000	1.000	0.283	2.967	0.390
EI-11-F-dG4sf	88	444.0	-752.0	-330.8	0.541	-0.678	0.117	0.310	3.914	0.265
JTT-ML91+-11-F-dG4sf	88	1226.6	-2317.2	-1896.0	1.495	0.358	0.098	0.373	2.350	0.442
WAG-ML91+-11-F-dG4sf	88	1290.2	-2444.5	-2023.3	1.339	0.220	0.116	0.375	3.544	0.390
KHG-ML200-11-F-dG4sf	88	1328.4	-2520.8	-2099.6	1.406	0.986	0.208	0.181	2.062	0.574
LG-ML91+-11-F-dG4sf	88	1360.7	-2585.4	-2164.2	0.992	0.122	0.123	0.278	3.769	0.416

^a^"-F" means that the equilibrium frequencies are estimated to be equal to those in the alignment; equal codon usage is assumed. "-dG*m*r" and "-dG*m*s" mean discrete gamma distributions with *m* categories of unequal probabilities for the rate variation and the variation of selective constraint across sites, respectively. "-dG*m*sf" means the equilibrium frequencies for respective categories are estimated from their posterior probabilities for sites. The number string in the model name indicates the number of parameters optimized for the substitution rate matrix, and the remaining strings denote a rate matrix or a selective constraint matrix used.

^b^The number of adjustable parameters.

^c^Difference from the reference state; Δ*ℓ* = *ℓ* + 122106.2, ΔAIC = AIC - 244252.3, and ΔBIC = BIC - 244376.2. The reference tree topology is Tree-6 in [34].

^d^$w_{\text{ab}}=\text{min}[{\beta w}_{\text{ab}}^{\text{estimate}}+w_{0}(1-\delta_{\text{ab}}),0]$; $w_{\text{ab}}^{\text{estimate}}$ is the one specified by the model name.

^e^The value parenthesized means that the parameter is fixed at the value specified.

^f^The average of $e^{w_{\text{ab}}}$ over all amino acid pairs {*a,b*}; $\langle e^{w_{\text{ab}}}\rangle \equiv \frac{1}{190}\sum_{a}\sum_{b>a}e^{w_{\text{ab}}}$.

^g^The ratio of double to single and of triple to double nucleotide change exchangeability; $\hat{m}\equiv {\hat{m}}_{\left[\text{tc}][\text{ag}\right]}$.

^h^The ratio of mean transitional to mean transversional exchangeability; ${\hat{m}}_{\text{tc}|\text{ag}}/{\hat{m}}_{\left[\text{tc}][\text{ag}\right]}$.

^i^The shape parameter of a discrete gamma distribution for the variation of mutation rate or selective constraint across sites.

**Table 4:** Comparisons between various amino acid and codon substitution models for the reference phylogenetic tree of the cpProt-55

**Substitution model**^**a**^	**K**^**b**^	**Δ*ℓ***^**c**^	**ΔAIC**^**c**^	**ΔBIC**^**c**^	***β***^**de**^	***w***_***0***_^**d**^	${\langle e^{w_{\text{ab}}}\rangle }^{f}$	${\hat{m}}^{g}$	^**h**^	${\hat{\alpha }}^{i}$
Amino acid substitution models										
cpREV64-dG4r	1	0.0	0.0	0.0						0.292
LG-F-dG4r	20	-9935.0	19908.0	20051.6						0.339
mtREV-F-dG4r	20	-7875.1	15788.1	15931.7						0.259
WAG-F-dG4r	20	-7649.6	15337.2	15480.7						0.348
FLU-F-dG4r	20	-5732.4	11502.7	11646.3						0.269
cpREV10-F-dG4r	20	-5649.9	11337.8	11481.3						0.349
JTT-F-dG4r	20	-4671.9	9381.8	9525.3						0.347
cpREV64-F-dG4r	20	-803.8	1645.6	1789.2						0.345
Mechanistic codon substitution models										
Equal-Constraint-10-F-dG4r	30	332.4	-606.8	-387.7	(0.0)	-0.000	1.000	0.109	2.556	0.287
EI-11-F-dG4r	31	565.0	-1070.0	-843.3	0.101	0.101	0.782	0.119	2.686	0.285
KHG-ML200-11-F-dG4r	31	1150.9	-2241.8	-2015.1	0.386	0.139	0.491	0.102	2.249	0.287
WAG-ML91+-11-F-dG4r	31	1164.8	-2269.5	-2042.9	0.334	0.065	0.475	0.161	2.648	0.286
LG-ML91+-11-F-dG4r	31	1179.4	-2298.7	-2072.0	0.271	0.165	0.548	0.139	2.666	0.286
JTT-ML91+-11-F-dG4r	31	1426.3	-2792.6	-2565.9	0.430	0.132	0.421	0.187	2.234	0.287
JTT-ML91+-11-F-dG8r	31	1666.2	-3272.3	-3045.6	0.435	0.134	0.418	0.182	2.237	0.295
Equal-Constraint-10-F-dG4s	30	346.8	-635.6	-416.4	(0.0)	-0.233	0.793	0.113	2.549	0.286
EI-11-F-dG4s	31	962.6	-1865.2	-1638.5	0.264	-0.255	0.341	0.135	2.727	0.262
KHG-ML200-11-F-dG4s	31	1472.2	-2884.3	-2657.7	0.434	-0.672	0.199	0.101	2.326	0.284
WAG-ML91+-11-F-dG4s	31	1632.9	-3205.8	-2979.1	0.607	-0.344	0.189	0.167	2.633	0.258
LG-ML91+-11-F-dG4s	31	1742.9	-3425.8	-3199.1	0.544	0.005	0.248	0.148	2.630	0.276
JTT-ML91+-11-F-dG4s	31	1886.9	-3713.7	-3487.1	0.788	0.221	0.235	0.191	2.198	0.253
JTT-ML91+-11-F-dG8s	31	2176.2	-4292.4	-4065.7	0.854	0.257	0.218	0.200	2.170	0.275
Equal-Constraint-10-F-dG4sf	87	1224.3	-2276.5	-1626.7	(0.0)	-0.174	0.840	0.115	2.537	0.276
EI-11-F-dG4sf	88	1920.6	-3667.2	-3009.8	0.279	-0.231	0.335	0.135	2.665	0.251
KHG-ML200-11-F-dG4sf	88	2105.0	-4036.1	-3378.7	0.455	-0.626	0.200	0.102	2.296	0.286
WAG-ML91+-11-F-dG4sf	88	2320.8	-4467.5	-3810.1	0.633	0.060	0.270	0.165	2.528	0.249
LG-ML91+-11-F-dG4sf	88	2369.0	-4564.0	-3906.7	0.523	-0.007	0.256	0.147	2.557	0.269
JTT-ML91+-11-F-dG4sf	88	2542.1	-4910.2	-4252.8	0.787	0.308	0.255	0.188	2.168	0.249

^a^"-F" means that the equilibrium frequencies are estimated to be equal to those in the alignment; equal codon usage is assumed. "-dG*m*r" and "-dG*m*s" mean discrete gamma distributions with *m* categories of unequal probabilities for the rate variation and the variation of selective constraint across sites, respectively. "-dG*m*sf" means the equilibrium frequencies for respective categories are estimated from their posterior probabilities for sites. The number string in the model name indicates the number of parameters optimized for the substitution rate matrix, and the remaining strings denote a rate matrix or a selective constraint matrix used.

^b^The number of adjustable parameters.

^c^Difference from the reference state; Δ*ℓ* = *ℓ*+ 217554.4, ΔAIC=AIC- 435110.9, and ΔBIC=BIC- 435118.5. The reference tree topology is the one reported in [35].

^d^$w_{\text{ab}}=\text{min}[\beta w_{\text{ab}}^{\text{estimate}}+w_{0}(1-\delta_{\text{ab}}),0]$; $w_{\text{ab}}^{\text{estimate}}$ is the one specified by the model name.

^e^The value parenthesized means that the parameter is fixed at the value specified.

^f^The average of $e^{w_{\text{ab}}}$ over all amino acid pairs {*a,b};*$\langle e^{w_{\text{ab}}}\rangle \equiv \frac{1}{190}\sum_{a}\sum_{b>a}e^{w_{\text{ab}}}$.

^g^The ratio of double to single and of triple to double nucleotide change exchangeability; $\hat{m}\equiv {\hat{m}}_{\left[\text{tc}][\text{ag}\right]}$.

^h^The ratio of mean transitional to mean transversional exchangeability; ${\hat{m}}_{\text{tc}|\text{ag}}/{\hat{m}}_{\left[\text{tc}][\text{ag}\right]}$.

^i^The shape parameter of a discrete gamma distribution for the variation of mutation rate or selective constraint across sites.

**Table 5:** Comparisons between various amino acid and codon substitution models for the reference phylogenetic tree of the HA_Human-Flu-A-H1N1

**Substitution model**^**a**^	**K**^**b**^	**Δ*ℓ***^**c**^	**ΔAIC**^**c**^	**ΔBIC**^**c**^	***β***^**de**^	***w***_***0***_^**d**^	${\langle e^{w_{\text{ab}}}\rangle }^{f}$	${\hat{m}}^{g}$	^**h**x^	${\hat{\alpha }}^{i}$
Amino acid substitution models										
FLU-dG4r	1	0.0	0.0	0.0						0.913
mtREV-F-dG4r	20	-985.5	2009.0	2085.2						0.809
LG-F-dG4r	20	-885.1	1808.3	1884.5						0.856
WAG-F-dG4r	20	-777.1	1592.2	1668.4						0.882
cpREV10-F-dG4r	20	-695.8	1429.5	1505.8						0.858
JTT-F-dG4r	20	-386.3	810.5	886.7						0.892
cpREV64-F-dG4r	20	-167.9	373.8	450.0						0.840
FLU-F-dG4r	20	8.1	21.7	98.0						0.907
Mechanistic codon substitution models										
Equal-Constraint-10-F-dG4r	30	203.4	-348.7	-232.4	(0.0)	-1.109	0.330	0.010	4.768	0.828
EI-11-F-dG4r	31	332.7	-605.4	-485.1	0.311	-0.609	0.212	0.013	4.835	0.880
LG-ML91+-11-F-dG4r	31	394.6	-729.2	-608.8	0.453	-0.690	0.151	0.014	4.792	0.920
WAG-ML91+-11-F-dG4r	31	405.2	-750.4	-630.1	0.565	-0.679	0.145	0.018	4.825	0.940
KHG-ML200-11-F-dG4r	31	410.0	-760.0	-639.7	0.676	-0.214	0.202	0.009	3.287	0.923
JTT-ML91+-11-F-dG4r	31	418.3	-776.6	-656.2	0.636	-0.425	0.162	0.027	3.725	0.923
JTT-ML91+-11-F-dG8r	31	441.2	-822.3	-702.0	0.641	-0.446	0.157	0.026	3.745	0.923
Equal-Constraint-10-F-dG4s	30	206.3	-354.7	-238.4	(0.0)	-1.434	0.238	0.010	4.754	0.823
EI-11-F-dG4s	31	328.5	-596.9	-476.6	0.332	-0.495	0.225	0.015	4.741	0.887
LG-ML91+-11-F-dG4s	31	397.6	-735.2	-614.9	0.454	-0.962	0.115	0.014	4.780	0.903
KHG-ML200-11-F-dG4s	31	412.5	-765.1	-644.7	0.676	-0.662	0.129	0.009	3.300	0.923
WAG-ML91+-11-F-dG4s	31	415.0	-770.0	-649.6	0.627	-0.303	0.190	0.021	4.620	0.890
JTT-ML91+-11-F-dG4s	31	421.1	-782.2	-661.9	0.635	-0.761	0.116	0.027	3.722	0.918
JTT-ML91+-11-F-dG8s	31	457.7	-855.4	-735.1	0.731	-0.317	0.152	0.029	3.630	0.911
Equal-Constraint-10-F-dG4sf	87	297.2	-422.3	-77.4	(0.0)	-1.549	0.212	0.010	4.603	0.716
EI-11-F-dG4sf	88	405.8	-637.7	-288.7	0.313	-0.526	0.229	0.014	4.366	0.856
KHG-ML200-11-F-dG4sf	88	428.1	-682.2	-333.2	0.565	-0.674	0.155	0.010	3.397	0.920
LG-ML91+-11-F-dG4sf	88	439.7	-705.5	-356.5	0.369	-1.050	0.128	0.016	4.575	0.885
WAG-ML91+-11-F-dG4sf	88	443.3	-712.6	-363.7	0.658	-0.012	0.241	0.023	4.446	0.864
JTT-ML91+-11-F-dG4sf	88	447.8	-721.6	-372.7	0.686	-0.200	0.185	0.032	3.520	0.871

^b^"-F" means that the equilibrium frequencies are estimated to be equal to those in the alignment; equal codon usage is assumed. "-dG*m*r" and "-dG*m*s" mean discrete gamma distributions with*m*categories of unequal probabilities for the rate variation and the variation of selective constraint across sites, respectively. "-dG*m*sf" means the equilibrium frequencies for respective categories are estimated from their posterior probabilities for sites. The number string in the model name indicates the number of parameters optimized for the substitution rate matrix, and the remaining strings denote a rate matrix or a selective constraint matrix used.

^b^The number of adjustable parameters.

^c^Difference from the reference state; Δ*ℓ* = *ℓ*+20059.7, ΔAIC=AIC-40121.5, and ΔBIC = BIC-40125.5. The reference tree topology is one inferred by FastTree-2 [36].

^d^$w_{\text{ab}}=\text{min}[\beta w_{\text{ab}}^{\text{estimate}}+w_{0}(1-\delta_{\text{ab}}),0]$; $w_{\text{ab}}^{\text{estimate}}$ is the one specified by the model name.

^e^The value parenthesized means that the parameter is fixed at the value specified.

^f^The average of $e^{w_{\text{ab}}}$ over all amino acid pairs {*a,b*}; $\langle e^{w_{\text{ab}}}\rangle \equiv \frac{1}{190}\sum_{a}\sum_{b>a}e^{w_{\text{ab}}}$.

^g^The ratio of double to single and of triple to double nucleotide change exchangeability; $\hat{m}\equiv {\hat{m}}_{\left[\text{tc}][\text{ag}\right]}$.

^h^The ratio of mean transitional to mean transversional exchangeability; ${\hat{m}}_{\text{tc}|\text{ag}}/{\hat{m}}_{\left[\text{tc}][\text{ag}\right]}$.

^i^The shape parameter of a discrete gamma distribution for the variation of mutation rate or selective constraint across sites.

The best models in the present amino acid substitution models for the respective datasets are cpREV64 for cpProt-55, mtREV for mammal-mtProt, and FLU for HA_Human-Flu-A-H1N1. This fact is expected because cpREV64 was estimated [28] from the full set of 77 protein-coding genes in the 64 chloroplast genomes including cpProt-55, mtREV [6] from the 12 mitochondrial proteins of 20 vertebrate species, and FLU [29] from ∼113000 influenza proteins including HA_Human-Flu-A-H1N1. Hence, the fact shown in the tables indicates that these matrices certainly represent substitution tendencies specific to the respective protein families. On the other hand, cpREV10 [8] was estimated from the smaller dataset than that for cpREV64, that is, 45 proteins in 9 chloroplast genomes including 5 land plants and the complete genome of cyanobacteria. The size of database used for cpREV10 may cause the cpREV10 to perform worse than JTT for cpProt-55.

These tables also show that the log-likelihood values for JTT, WAG, and LG are much smaller than those of the reference models and also differ largely from one another, indicating that these empirical substitution matrices represent the average tendencies of substitutions over various protein families but lack gene-level resolution. What are the characteristics of substitutions specific to each protein family? Certainly the strength of selective constraint against amino acid substitutions depends on the type of protein, and varies across sites in a protein. However, the dependence of selective constraint on the substituted type of amino acid may result primarily from amino acid properties. Hence, in the present mechanistic codon substitution model, we approximate the selective constraint (*w*_*ab*_) for a target protein family with a linear function of the particular value ( $w_{\text{ab}}^{\text{estimate}}$) that was estimated from an empirical amino acid substitution frequency matrix and represents the average strength of selective constraint against each type of amino acid substitution over various proteins. In addition, the tendency of nucleotide mutation may differ among nuclear, mitochondrial and chloroplast DNA, and selection on nucleotide substitutions at the DNA/RNA level may exist and depend on each gene. In the present model, a nucleotide mutation rate matrix is fully parameterized by 8 parameters, and one additional parameter (*m*) is employed to represent the ratio of multiple to single nucleotide changes in a codon.

Tables 3, 4, and 5 clearly show that all the codon substitution models together with the respective selective constraint matrices estimated from JTT, LG, WAG, and KHG significantly outperform the best amino acid substitution model in all the three datasets, even though 11 more parameters must be estimated. Here we should notice that the best amino acid substitution rate matrices were estimated from the protein families corresponding to the respective target proteins. In addition, it is important to notice that in agreement with common biological knowledge the mean transitional exchangeability is estimated to be far larger than the mean transversional exchangeability for all protein families in all codon substitution models.

As already claimed for codon sequences in [27], the variation of selective constraint across sites (dG4s) is a better model than the variation of codon mutation rate (dG4r) in all the three datasets. This fact confirms a common presumption that substitution rate variations across sites detected by amino acid substitution models are caused primarily by the variation of selective constraint rather than the mutation rate variation.

In the present analysis, 4 categories of unequal probabilities are employed to represent a Γ distribution. This number of categories is chosen to be not sufficient but minimum to represent a Γ distribution. Actually, as shown in Tables 3, 4, and 5, representing a Γ distribution by 8 categories of unequal probabilities can significantly improve the log-likelihood.

In Tables 3, 4, and 5, the results for the equal constraint model are also listed; selective constraint is the same for all types of amino acid substitutions, that is, *β* = 0. The AIC and BIC values of the equal constraint model are larger for the mammalian-mtProt but smaller for the cpProt and HA_Human-Flu-A-H1N1 than those of the reference amino acid substitution models. Accordingly, the estimated value of the slope *β* of a linear function for selective constraints, is larger for the mammalian-mtProt than for the cpProt and HA_Human-Flu-A-H1N1. This fact indicates that the dependence (*β*) of selective constraint on amino acid type is less effective for both cpProt and HA_Human-Flu-A-H1N1, although this may result from a property that both the datasets consist of relatively closely-related sequences and contain mostly conservative amino acid substitutions.

In the Energy-Increment-based (EI) model shown in Tables 3, 4, and 5, the selective constraint matrix used is the one estimated on the basis of the mean increment of contact energy and residue-volume change accompanied by an amino acid replacement [26]. Although the energy-increment-based selective constraints perform better than the equal constraint, it does not perform as well as the empirical selective constraints estimated from the JTT, WAG, LG, and KHG, indicating the good quality of their empirical values of the selective constraints.

In the present discrete gamma distribution model for the variation of selective constraint, the value of *w*_**0**_ differs among categories, but the same equilibrium frequencies of amino acids, which are estimated to be equal to those in the alignment, are employed for all the categories. Amino acid frequencies strongly depend on residue location in protein structures. Typically, non-polar residues are more and polar residues are less frequent in the interior of protein structures, where selective constraint against amino acid replacements tends to be more restrictive [27]**,**[40]**,**[41]. There must be a correlation between the strength of selective constraint and the equilibrium frequencies of amino acids. Hence, different equilibrium frequencies should be employed in principle for each category. Substitution rate matrices that differ only in their equilibrium frequencies were employed in [42]**,**[43]. In [44]**,**[45], a different rate matrix was employed for each of gamma rate categories and biochemical and structural categories. Le et al. [46] estimated from a very large alignment database and then tested four amino acid substitution rate matrices each of which corresponds to one discrete gamma category or one distribution-free category and has different exchangeabilities and equilibrium frequencies. Here, we estimate amino acid frequencies for each category from the posterior probabilities of sites being at each category and then parameters are optimized again with the estimated amino acid frequencies for each category. The AIC and BIC values for this new model named with a suffix "-dG4sf" are also listed in Tables 3, 4, and 5. The values of AIC and BIC are improved for mammal-mtProt and cpProt-55 but not for HA_Human-Flu-A-H1N1. This model requiring additional 57 parameters needs a sufficient number of amino acid substitutions in an alignment to yield better values of AIC and BIC.

## Conclusions

The greatest advantage of employing a mechanistic codon substitution model over amino acid substitution models resides in an intrinsic property that mutational tendencies of codons and selective constraints against amino acid changes can be separately formulated in codon substitution models. As a result, codon mutational tendencies and the strength of selective constraint can be tailored to those in each gene with the additional 11 parameters, although a sufficient number of sequences and more calculations are needed for their estimation. Also, besides mutation rate variation, the variation of selective constraint across sites can be taken into account. At the amino acid level, synonymous substitutions cannot be identified. Even so, taking account of synonymous substitutions with a codon substitution model improves its performance, as shown in the present analysis. Since mutations occur in nucleotide level, codon mutations may be well approximated by a Markov process. However, Markovian properties are lost in the process of amino acid substitutions, because of redundancy of translation to amino acids. A hidden Markov model, in which codon types are hidden states changing in a Markov process, is more appropriate to represent amino acid substitutions. This may be one of reasons why the mechanistic codon substitution model outperforms any amino acid substitution model examined here.

A conclusion is drawn that the mechanistic codon substitution model is superior to amino acid substitution models even for protein sequences in evolutionary analysis.

## Methods

### Likelihood of amino acid sequences in a codon substitution model

Given a phylogenetic tree *T* and a codon substitution model Θ, in which codon substitutions are assumed to occur independently at each site and to be in the stationary state of a time-reversible Markov process, the conditional probability *P*(***A***|*T*,Θ) that an alignment *A* ≡ {***A***_**1**_,***A***_2_,…,***A***_*L*_} with *L* sites is observed is represented as the product over sites of those of the alignments ***A***_*i*_ ≡ {*A*_1*i*_,*A*_2*i*_,…,*A*_*Ni*_}^**′**^ at site *i*; $P(A|T,\Theta )=\prod_{i}P(A_{i}|T,\Theta )$. The likelihood of the phylogenetic tree *T* and the model Θ for the alignment at each site can be calculated as

(1) $$\begin{array}{l} P(A_{i}|T,\Theta )=\underset{\mu }{\sum }\underset{\nu }{\sum }\delta_{A_{\ell i}a_{\nu }}P(\nu |\mu ,t_{\ell },\Theta )f_{\mu }P_{v_{\ell }}(A_{i}|v_{\ell }=\mu ,T,\Theta ) \end{array}$$

where *v*_*ℓ*_ is the ancestor node connected to a leaf node *ℓ* with branch length *t*_*ℓ*_, *μ* and *ν* denote the type of codon, and *f*_*μ*_ is the equilibrium frequency of codon *μ*. The *P*(*ν*|*μ*,*t*_*ℓ*_,Θ) is the substitution probability from *μ* to *ν* in the time interval *t*_*ℓ*_, and $P_{v_{\ell }}(A_{i}|v_{\ell }=\mu ,T,\Theta )$ is the likelihood of the parent subtree with the node *v*_*ℓ*_ = *μ* connected to the leaf node *ℓ*. The $\delta_{A_{\ell i}a_{\nu }}$, which represents a code table, is the Kronecker delta and takes one if *A*_*ℓi*_ = *a*_*ν*_ otherwise zero, where *a*_*ν*_ is the type of amino acid corresponding to codon *ν*. We simply assumed equal codon usage here to estimate the equilibrium frequencies of codons from the amino acid composition in the alignment.

The posterior frequency of amino acid *a* in the category *θ*_*α*_ of rate or selective constraint is calculated with

(2) $$f_{a}(\theta_{\alpha })\propto \underset{i}{\sum }\underset{s}{\sum }\delta_{A_{\text{si}}a}P(\theta_{\alpha }|A_{i},\hat{T},\hat{\Theta })$$

where $\hat{T}$** and**$\hat{\Theta }$ denote their estimates, and the posterior probability of site *i* being at the category *θ*_*α*_ is

(3) $$P(\theta_{\alpha }|A_{i},\hat{T},\hat{\Theta })=\frac{P(A_{i}|\hat{T},\hat{\Theta },\theta_{\alpha })P(\theta_{\alpha })}{P(A_{i}|\hat{T},\hat{\Theta })}$$

The posterior frequencies of amino acids for each category may be used in the next run as the equilibrium frequencies for each category.

### A mechanistic codon substitution model with multiple nucleotide changes

We assume that substitutions from *μ* to *ν* occur with a constant substitution rate *R*_*μν*_ per unit time and the detailed balance condition between equilibrium states; hence, *P*(*ν*|*μ*,*t*,Θ) = exp(*R*(Θ)*t*) with *R*_*μν*_ = *r*_*μν*_*f*_*ν*_ and *r*_*μν*_ = *r*_*νμ*_. The unit of time is chosen in such a way that the total substitution rate is equal to 1; $-\sum_{\mu }f_{\mu }R_{\mu \mu }=1$. Thus, only relative values among the exchangeability *r*_*μν*_ are meaningful.

In the mechanistic codon substitution model [26,27], the substitution rate *R*_*μν*_ is formulated as the product, *R*_*μν*_ ∝ *M*_*μν*_*F*_*μν*_ for *μ* ≠ *ν*, of a mutation rate *M*_*μν*_ and the average ratio of fixation *F*_*μν*_ that is represented as $F_{\mu \nu }=(f_{\nu }/f_{\nu }^{\text{mut}})e^{w_{\mu \nu }}$, where $f_{\nu }^{\text{mut}}$ is the equilibrium codon frequencies of mutation (*M*), and *w*_*μν*_(= *w*_*νμ*_) represents selective constraint on mutations between *μ* and *ν*. We assume the selective pressure appears primarily on an amino acid sequence; if *μ* or *ν* ∈ {stop codons}, then *w*_*μν*_ = -*∞* otherwise $w_{\mu \nu }=w_{a_{\mu }b_{\nu }}$, where *a*_*μ*_ and *b*_*ν*_ are the amino acid types encoded by the codons *μ* and *ν*, respectively. A code table specific to each gene such as the standard and vertebrate mitochondrial code tables is employed. No selection is assumed for synonymous substitutions; *w*_*ab*_ = 0 for *a* = *b*. We estimated *w*_*ab*_ by fitting a substitution probability matrix to each empirical amino acid substitution frequency matrix such as JTT with a maximum likelihood method [26]. Because the strength of selective constraint on amino acid substitutions depends strongly on the type of protein, we approximate the selective constraint for a target protein by a linear function of that estimated from an empirical amino acid substitution frequency matrix;

(4) $$w_{\text{ab}}=\min\;\left[{\beta w}_{\text{ab}}^{\text{estimate}}+w_{0}(1-\delta_{\text{ab}}),\;0\right]$$

where negative selection on amno acid replacements is assumed. Positive selection will be taken into account if selective constraints are variable over sites. The variation of selective constraint is approximated by a discrete gamma distribution [38]**,**[39] in which a given number of categories represent a Γ distribution with unequal probabilities virtually to increase the number of categories. For details including the discrete gamma representation for the variations of selective constraint and also mutation rate, see Additional file 1.

We represent the codon mutation rate matrix *M* as follows by assuming that nucleotide mutations occur independently of codon positions but multiple nucleotide changes can infinitesimally occur.

(5) $$M_{\mu \nu }\equiv \overset{3}{\underset{i=1}{\prod }}\left[\delta_{\mu_{i}\nu_{i}}+(1-\delta_{\mu_{i}\nu_{i}})m_{\mu_{i}\nu_{i}}f_{\nu_{i}}^{\text{mut}}\right]\;\text{for}\;\mu \neq \nu$$

where $m_{\mu_{i}\nu_{i}}(=m_{\nu_{i}\mu_{i}})$ is a mutation exchangeability matrix between the four types of nucleotides, $f_{\nu_{i}}^{\text{mut}}$ is the mutation equilibrium frequency of nucleotide *ν*_*i*_, $\delta_{\mu_{i}\nu_{i}}$ is the Kronecker’s *δ*, and the index *μ*_*i*_ denotes the *i*th nucleotide in the codon *μ*; *μ* = (*μ*_1_,*μ*_2_,*μ*_3_) where *μ*_*i*_ ∈ { a, t, c, g }, and $f_{\nu =(\nu_{1},\nu_{2},\nu_{3})}^{\text{mut}}=f_{\nu_{1}}^{\text{mut}}f_{\nu_{2}}^{\text{mut}}f_{\nu_{3}}^{\text{mut}}$. The matrix (*m*_*μν*_) is parameterized with 9 parameters; one additional parameter (*m*) is needed to represent the ratio of multiple to single nucleotide changes. See Additional file 1 for details.

### Protein sequence data used

Amino acid and codon substitution models are evaluated by using the following three datasets of protein sequences. 

1. mammalian-mtProt, which consists of fast-evolving interspecific mitochondrial protein sequences concatenating 12 protein-coding genes from 69 mammalian species [34]. The alignments of the genes, each of which was made with the codon sequences by the modified version [27] of the ClustalW2 [47], consist of 3618 sites. The tree topology estimated as Tree-6 by [34] is used here as the most probable one. Overlapped segments between genes were removed from protein sequences.

2. cpProt-55, which consists of closely-related chloroplast-encoded protein sequences concatenating 52 protein-coding genes from 55 chloroplast genomes of the major angiosperm lineages out of the 64 taxa analyzed in [35]. The tree topology reported in [35] is assumed as the most probable one in the present analysis. The alignments of the genes, each of which was made with the codon sequences by the modified version [27] of the ClustalW2 [47], consist of 14128 sites. The cpREV64 [28] was estimated from the full set of 77 protein-coding genes in the 64 genomes.

3. HA_Human-Flu-A-H1N1, which consists of fast-evolving hemagglutinin proteins of Human influenza A; relatively-dissimilar 1309 sequences out of 4231 sequences of HA protein from the H1N1 type of human influenza A in the NCBI entire influenza database. These sequences were aligned by the MAFFT version 7 with the FFT-NS-2 option [48], and the tree topology assumed as the most probable one is the one inferred by the FastTree version 2 [36] with the JTT and CAT options. In the present analysis, 408 sites, which do not include deletions, out of 595 sites in the multiple sequence alignment are used, because sites with gaps were excluded in the estimation of FLU [29].

These datasets are chosen, because the empirical amino acid substitution rate matrices, cpREV64 [28], mtREV [6], and FLU [29], that were designed as those specific to the respective protein sequences are available.

## Competing interests

The author declares that he has no competing interests.

## Supplementary Material

Additional file 1 **Methods.** A PDF file in which the details of the methods are described. Click here for file
