# Supplementary material for: Superiority of a mechanistic codon substitution model even for protein sequences in Phylogenetic analysis
Source: BMC Evol Biol. 2013 Nov 21;13:257. doi: 10.1186/1471-2148-13-257 (PMC4225520; doi:10.1186/1471-2148-13-257)
Supplement: Additional file 1 — Methods. A PDF file in which the details of the methods are described. [file 1471-2148-13-257-S1.pdf]

RESEARCH

Supplement for
Superiority of a Mechanistic Codon Substitution Model Even for Protein Sequences in Phylogenetic Analysis

Sanzo Miyazawa

Correspondence:
sanzo.miyazawa@gmail.com
6-5-607 Miyanodai,
Sakura, Chiba 285-0857, Japan
Full list of author information is available at the end of the article

BMC Evolutionary Biology **13**, 257, 2013.

(11/26/2013)

Methods

Likelihood of amino acid sequences in a codon substitution model

Given a phylogenetic tree T and a codon substitution model Θ, in which codon substitutions are assumed to occur independently at each site, the conditional probability $P(\boldsymbol{A}|T,\Theta)$ that an alignment $A \equiv (\boldsymbol{A}_1, \boldsymbol{A}_2, \ldots, \boldsymbol{A}_L)$ of N sequences with L sites is observed is represented as the product over sites of those of the alignments $\boldsymbol{A}_i \equiv (A_{1i}, A_{2i}, \ldots, A_{Ni})'$ at site i.

$$P(A|T,\Theta) \quad = \quad \prod_i P(\boldsymbol{A}_i|T,\Theta) \tag{1}$$

The likelihood of the phylogenetic tree T and the model Θ for the alignment at each site can be calculated as

$$P(\boldsymbol{A}_i|T,\Theta) \quad = \quad \sum_{\theta_\alpha} P(\boldsymbol{A}_i|T,\Theta,\theta_\alpha)P(\theta_\alpha) \tag{2}$$

where $P(\theta_\alpha)$ is the *a priori* probability distribution of a parameter θ_α for the variation of selective constraint or mutation rate across sites [1, 2]. Here, a mechanistic codon substitution model [1, 2] is used as the evolutionary model Θ. Then, if substitutions are assumed to be in the equilibrium state of a time-reversible Markov process, the likelihood of a sequence alignment $\boldsymbol{A}_i$ for site i will be calculated by taking any node as a root node. Let us assume here that the root node is the node v_ℓ connected to a leaf node ℓ with branch b_ℓ.

$$P(\boldsymbol{A}_i|T,\Theta,\theta_\alpha) \quad = \quad \sum_\mu \sum_\nu \delta_{A_{\ell i} a_\nu} P(\nu|\mu, t_\ell, \Theta, \theta_\alpha) f_\mu P_{v_\ell}(\boldsymbol{A}_i|v_\ell = \mu, T, \Theta, \theta_\alpha) \tag{3}$$

where μ and ν denote the type of codon, and f_μ is the equilibrium frequency of μ. $P(\nu|\mu, t_\ell, \Theta, \theta_\alpha)$ is a substitution probability from μ to ν at the branch b_ℓ whose length is equal to t_ℓ. $P_{v_\ell}(\boldsymbol{A}_i|v_\ell = \mu, T, \Theta, \theta_\alpha)$ is the likelihood of the parent subtree with the node $v_\ell = \mu$ connected to the leaf node ℓ. $\delta_{A_{\ell i} a_\nu}$ is the Kronecker delta and takes one if $A_{\ell i} = a_\nu$ otherwise zero, where a_ν is the type of amino acid corresponding to codon ν. Equal codon usage is simply assumed here to estimate the equilibrium frequencies of codons from the amino acid composition in the alignment.

In the maximum likelihood (ML) method for a phylogenetic tree, the tree T and parameters Θ are estimated by maximizing the likelihood.

$$(\hat{T}, \hat{\Theta}) \quad \equiv \quad \arg\max_{T,\Theta} P(A|T,\Theta) \tag{4}$$

The posterior probability of site i being at the category θ_α is

$$P(\theta_\alpha|\boldsymbol{A}_i, \hat{T}, \hat{\Theta}) \quad = \quad \frac{P(\boldsymbol{A}_i|\hat{T}, \hat{\Theta}, \theta_\alpha)P(\theta_\alpha)}{P(\boldsymbol{A}_i|\hat{T}, \hat{\Theta})} \tag{5}$$

Then, the posterior frequencies of amino acid a in the category θ_α is calculated with

$$f_a(\theta_\alpha) \quad \propto \quad \sum_i \sum_s \delta_{A_{si} a} P(\theta_\alpha | \boldsymbol{A}_i, \hat{T}, \hat{\Theta}) \tag{6}$$

from which codon frequencies for the category are estimated with the assumption of equal codon usage. The posterior frequencies of amino acids for each category may be used in the next run as the equilibrium frequencies for each category.

A mechanistic codon substitution model with multiple nucleotide changes

When substitutions independently occur at each site with a constant substitution rate $R_{\mu\nu}$ per unit time from codon μ to ν, the substitution probability matrix $P(\nu|\mu, t, \Theta, \theta_\alpha)$ during time t is calculated as

$$P(\nu|\mu, t, \Theta, \theta_\alpha) \quad = \quad \exp(R(\Theta, \theta_\alpha) t) \tag{7}$$

Assuming that the detailed balance condition between states is satisfied, i.e., $f_\mu R_{\mu\nu} = f_\nu R_{\nu\mu}$, the substitution rate $R_{\mu\nu}$ is represented as

$$R_{\mu\nu} \quad = \quad r_{\mu\nu} f_\nu \quad , \quad r_{\mu\nu} = r_{\nu\mu} \quad \text{for } \mu \neq \nu \tag{8}$$

where f_μ is the equilibrium composition; $\sum_\mu f_\mu R_{\mu\nu} = 0$. The symmetric matrix r is named an exchangeability matrix. In the case of the codon substitution matrix, the equilibrium frequencies of stop codons are set to be equal to 0, and therefore the probability flow from any to a stop codon and its inverse flow are always equal to 0. The unit of time is chosen in such a way that the total rate of R is equal to 1;

$$\sum_\mu f_\mu \sum_{\nu \neq \mu} R_{\mu\nu} \quad = \quad -\sum_\mu f_\mu R_{\mu\mu} = 1 \tag{9}$$

Therefore, only relative values among exchangeabilities $r_{\mu\nu}$ are meaningful.

In the present mechanistic codon substitution model [1, 2], the substitution rate $R_{\mu\nu}$ is represented as the product of a mutation rate $M_{\mu\nu}$ and the average ratio of fixation $F_{\mu\nu}$, which is defined to be the average fixation probability multiplied by the chromosomal population size, for mutations from codon μ to ν under selective pressure; $R_{\mu\nu} \propto M_{\mu\nu} F_{\mu\nu}$ for $\mu \neq \nu$. The M is also assumed to satisfy the detailed balance condition; $f_\mu^{\text{mut}} M_{\mu\nu} = f_\nu^{\text{mut}} M_{\nu\mu}$, where f_ν^{mut} is the equilibrium codon composition of the rate matrix M. Under this assumption, the average fixation ratio $F_{\mu\nu}$ must be represented as the product of the two terms, $f_\nu / f_\nu^{\text{mut}}$ and $e^{w_{\mu\nu}}$, where $w_{\mu\nu} = w_{\nu\mu}$; $F_{\mu\nu} = (f_\nu / f_\nu^{\text{mut}}) e^{w_{\mu\nu}}$ for $\mu \neq \nu$. Then, the exchangeability $r_{\mu\nu}$ can be represented as

$$R_{\mu\nu} \quad = \quad r_{\mu\nu} f_\nu = C_{\text{onst}} \, M_{\mu\nu} \frac{f_\nu}{f_\nu^{\text{mut}}} e^{w_{\mu\nu}} \quad \text{for } \mu \neq \nu \tag{10}$$

The arbitrary scaling constant C_{onst} is determined by Eq. 9.

The frequency-dependent term $f_\nu/f_\nu^{\rm mut}$ represents the effects of selective pressure at the DNA level as well as at the amino acid level, which change the codon frequency from the mutational equilibrium frequency $f_\nu^{\rm mut}$ to the frequency f_ν specific to a gene. The ratio of fixation F was explicitly given as a function of the fitnesses of mutants μ and ν [3, 4]. It must be equal to 0 for lethal mutations and equal to 1 for neutral mutations. Here, we approximate the average quantity $e^{w_{\mu\nu}}$ over mutants to be independent of codon frequencies. This quantity $e^{w_{\mu\nu}}$ is essentially the same as the one called the rate of acceptance by Miyata et al.[5]. We assume that selective pressure against codon replacements appears primarily on an amino acid sequence encoded by a nucleotide sequence; in other words, $w_{\mu\nu}$ for codon pair (μ,ν) depends only on the encoded amino acids a_μ and b_ν.

$$e^{w_{\mu\nu}} \equiv \begin{cases} e^{w_{a_\mu b_\nu}} & \text{for} \quad \mu,\nu \notin \{ \text{ stop codons } \} \text{ and } \mu \neq \nu \\ 0 & \text{for} \quad \mu \text{ or } \nu \in \{ \text{ stop codons } \} \text{ and } \mu \neq \nu \end{cases} \tag{11}$$

A code table specific to each gene such as the standard and vertebrate mitochondrial code tables is employed. At the amino acid level, there should be no selective pressure against synonymous mutations. Thus, the w_{ab} satisfies

$$w_{ab} = w_{ba} \quad , \quad w_{aa} = 0 \tag{12}$$

Selective constraints w_{ab} for a specific protein family are approximated with a linear function of a given estimate $w_{ab}^{\rm estimate}$;

$$w_{ab} = \min[\beta w_{ab}^{\rm estimate} + w_0(1-\delta_{ab}), 0] \tag{13}$$

where $w_{ab}^{\rm estimate}$ with
"estimate" $\in$ {EI, JTT-ML91+, WAG-ML91+, LG-ML91+, KHG-ML200} means the estimate of w_{ab}, which is a physico-chemical estimate based on the Energy-Increment-based (EI) method [1], or a ML estimate [1] from the empirical substitution frequency matrix of JTT, WAG, LG, or KHG. The value of w_{ab} is non-positive, assuming that on average there is negative selection on amino acid replacements; of course, $w_{ab}^{\rm estimate} \leq 0$ [2]. Positive selection will be taken into account if selective constraints are variable across sites. The parameter β, which is non-negative, adjusts the strength of selective constraint for a given protein family. The parameter w_0 directly controls the ratio of nonsynonymous to synonymous substitution exchangeability. The model with $\beta = 0$ is called the Equal-Constraint model, and the Equal-Constraint model with $w_0 = 0$ is called the No-Constraint model, which is equivalent to a nucleotide substitution model.

In the model EI, $w_{ab}^{\rm EI} \equiv -(\Delta\hat{\varepsilon}_{ab}^{\rm c} + \Delta\hat{\varepsilon}_{ab}^{\rm v})$, where $\Delta\hat{\varepsilon}_{ab}^{\rm c}$ and $\Delta\hat{\varepsilon}_{ab}^{\rm v}$ represent the effects of the mean increment of contact energies between residues and of residue-volume change due to an amino acid replacement, respectively; for details, see Supporting Information, Text S1, in [1]. The selective constraint matrices $w^{\rm estimate}$ with "estimate" $\in$ {JTT-ML91+, WAG-ML91+, LG-ML91+} are those estimated by maximizing the respective likelihoods of the 1-PAM amino acid substitution frequency matrices of JTT, WAG, and LG in the ML-91+ model [1]. Similarly, the

matrix $w^{\text{KHG-ML200}}$ was estimated from the 1-PAM KHG codon substitution frequency matrix in the ML-200 model [1]. These estimates of selective constraints are available as Supporting Information, Data S1, in [1]. These models are called here by the name of a selective constraint matrix with a suffix (n) meaning the number of adjustable parameters such as Equal-Constraint-n, EI-n, JTT/WAG/LG-ML91+-n, and KHG-ML200-n.

We represent the codon mutation rate matrix M in terms of nucleotide mutation rates as follows by assuming that nucleotide mutations occur independently of codon positions and multiple nucleotide changes can infinitesimally occur.

$$M_{\mu\nu} \equiv \prod_{i=1}^{3} [\delta_{\mu_i\nu_i} + (1 - \delta_{\mu_i\nu_i}) B_{i,\mu_i\nu_i}] \text{ for } \mu \neq \nu \tag{14}$$

where B_i is a mutation rate matrix between the four types of nucleotides at the ith codon position, $\delta_{\mu_i\nu_i}$ is the Kronecker's δ, and the index μ_i means the ith nucleotide in the codon μ; $\mu = (\mu_1, \mu_2, \mu_3)$ where $\mu_i \in$ { a, t, c, g } . Assuming that the rate matrix B_i satisfies the detailed balance condition, it is represented as

$$B_{i,\mu_i\nu_i} = m_{i,\mu_i\nu_i} f^{\text{mut}}_{i,\nu_i} \quad \text{for } i = 1, 2, 3 \tag{15}$$

$$m_{i,\mu_i\nu_i} = m_{i,\nu_i\mu_i} \tag{16}$$

$$f^{\text{mut}}_{\nu=(\nu_1,\nu_2,\nu_3)} = f^{\text{mut}}_{1,\nu_1} f^{\text{mut}}_{2,\nu_2} f^{\text{mut}}_{3,\nu_3} \tag{17}$$

where f^{mut}_{i,ν_i} is the equilibrium composition of nucleotide ν_i at the ith codon position, and $m_{i,\mu_i\nu_i}$ is the exchangeability between nucleotides μ_i and ν_i at the ith codon position. Because the B_i is assumed to satisfy the detailed balance condition, the M also satisfies the detailed balance condition.

If multiple nucleotide changes were completely ignored, then Eq. 14 would be simplified as $M_{\mu\nu} = ((1 - \delta_{\mu_1\nu_1}) B_{1,\mu_1\nu_1} \delta_{\mu_2\nu_2} \delta_{\mu_3\nu_3}) + (\delta_{\mu_1\nu_1}(1 - \delta_{\mu_2\nu_2}) B_{2,\mu_2\nu_2} \delta_{\mu_3\nu_3}) + (\delta_{\mu_1\nu_1} \delta_{\mu_2\nu_2}(1 - \delta_{\mu_3\nu_3}) B_{3,\mu_3\nu_3})$, whose formulation for a codon mutation rate matrix with Eq. 15 is the same as the one proposed by Muse and Gaut[6]. Here, it should be noted that $B_{i,\mu_i\nu_i}$ in Eq. 15 is defined to be proportional to the equilibrium nucleotide composition f^{mut}_{i,ν_i}. Alternatively, one may define $M_{\mu\nu}$ as $M_{\mu\nu} = \prod_{i=1}^{3} [\delta_{\mu_i\nu_i} + (1 - \delta_{\mu_i\nu_i}) m_{i,\mu_i\nu_i}] f^{\text{mut}}_{\nu}$ in the same way as Miyazawa and Jernigan[7] and others [8, 9] defined it to be proportional explicitly to the composition of the base triplet, f^{mut}_{ν}. This alternative definition with Eqs. 9 and 10 is equivalent to Eqs. 14 and 15 with $f^{\text{mut}}_{\nu_i} = 0.25$ and $m_{i,\mu_i\nu_i} \Rightarrow 4m_{i,\mu_i\nu_i}$, and thus it is a special case in the present formulation.

The No-Constraint model, in which there is no selective pressure on amino acid replacements ($w_{ab} = 0$), is a nucleotide substitution model extended to allow multiple nucleotide changes in infinitesimal time. Also, it is useful to note that the present model in the special case of $M_{\mu\nu}$ = constant becomes equivalent to an amino acid substitution model converted into a codon substitution model; if $(m_i)_{\mu_i\nu_i} = 4$ and $f^{\text{mut}}_{i,\nu_i} = 0.25$, then $M_{\mu\nu} = 1$ and Eq. 10 will become $r_{\mu\nu} \propto e^{w_{\mu\nu}}$ and equivalent to Eq. 4 in [2] with $r^{\text{empirical}}_{ab} \propto e^{\beta w^{\text{estimate}}_{ab}}$.

In the present analyses, we assume for simplicity that $m_{i,\mu_i\nu_i}$ and f^{mut}_{i,ν_i} do not depend on codon position i; that is, $m_{i,\xi\eta} = m_{\xi\eta}$ and $f^{\text{mut}}_{i,\xi} = f^{\text{mut}}_{\xi}$, where $\xi, \eta \in$

$\{a, t, c, g\}$. This approximation is reasonable because mutational tendencies may be independent of nucleotide position in a codon. Let us define $m_{[tc][ag]}$ to represent the average of the exchangeabilities of the transversion type, m_{ta}, m_{tg}, m_{ca}, and m_{cg}, and likewise $m_{tc|ag}$ to represent the average of the exchangeabilities of the transition type, m_{tc} and m_{ag}. We use the ratios $\{m_{\xi\eta}/m_{[tc][ag]}\}$ as parameters for exchangeabilities, and $m(\equiv m_{[tc][ag]})$ to represent the ratio of the exchangeability of double nucleotide change to that of single nucleotide change and also the ratio of the exchangeability of triple nucleotide change to that of double nucleotide change; note that the exchangeabilities of single, double, and triple nucleotide changes are of $O(m_{[tc][ag]}), O(m^2_{[tc][ag]})$, and $O(m^3_{[tc][ag]})$ in Eq. 14, respectively, and that Eq. 9 must be satisfied. Then, multiple nucleotide changes in infinitesimal time can be completely neglected by making the parameter $m(\equiv m_{[tc][ag]})$ approach zero with keeping $\{m_{\xi\eta}/m_{[tc][ag]}\}$ constant in Eq. 9. Also, it is noted that unlike the SDT model [10] double nucleotide changes at the first and the third positions in a codon are assumed to occur as frequently as doublet changes.

The number of parameters except equilibrium codon frequencies in the mechanistic codon substitution model is equal to 11; they are β, w_0, $m(\equiv m_{[tc][ag]})$, $m_{tc|ag}/m_{[tc][ag]}$, $m_{ag}/m_{tc|ag}$, $m_{ta}/m_{[tc][ag]}$, $m_{tg}/m_{[tc][ag]}$, $m_{ca}/m_{[tc][ag]}$, f_a^{mut}, f_c^{mut}, and f_g^{mut}, and fixed at certain values or optimized as ML parameters.

Variations of mutation rate and of selective constraint across codon sites

Taking account of the variation of amino acid substitution rate across sites always significantly increases the maximum likelihood of a phylogenetic tree in the analysis of amino acid sequences [11]. The variation of amino acid substitution rate can be caused by the variation of codon mutation rate and also by the variation of selective constraint on amino acids.

The variation of codon mutation rate across codon sites is also assumed to obey a Γ distribution [11] with a shape parameter α and the mean equal to 1, which is then approximated by a discrete-gamma distribution [12, 13] with m categories. Basically, categories, $0 < \Gamma_1 < \Gamma_2 < \ldots < \Gamma_m$, with equal probability are employed for the variations of rate, but if $\Gamma_2 < 0.1$, two categories of Γ_1 and Γ_2 will be merged and replaced by their (weighted) mean category of the sum of their probabilities, and the largest value of Γ_i, Γ_m, will be divided into two categories of the half of its probability, virtually increasing the number of categories; that is, each category has unequal probability. The shape parameter α is optimized as one of ML parameters. This model is specified with a suffix dGmr where m denotes the number of categories.

The variation of selective constraint across amino acid sites is approximated to obey a discrete-gamma distribution, too; the average of selective constraints over amino acid pairs (the mean acceptance rate), $\sum_a \sum_{b>a} e^{w_{ab}}/190$, is assumed to vary in a discrete-gamma distribution. The rate matrix of each category is scaled in such a way that the mean rate matrix with the prior probabilities of categories satisfies Eq. 9. The shape parameter α of the discrete-gamma distribution is optimized as one of ML parameters. This model is specified with a suffix dGms where m denotes the number of categories.

In the mechanistic codon substitution model, selective constraint $w_{i,ab}$ for ith category in a discrete-gamma distribution is calculated to satisfy the following equations.

$$\sum_i \Gamma_i p(\Gamma_i) = \frac{1}{190}\sum_a \sum_{b>a} e^{w_{ab}} \tag{18}$$

$$\frac{1}{190}\sum_a \sum_{b>a} e^{w_{i,ab}} = \Gamma_i \tag{19}$$

$$e^{w_{i,ab}} \equiv \begin{cases} \min[\exp(\beta w_{ab}^{\text{estimate}} + (w_0 + \log \gamma_i)(1-\delta_{ab})), 1] & \text{for } \Gamma_i < 1 \\ \Gamma_i(1-\delta_{ab}) + \delta_{ab} & \text{for } \Gamma_i \geq 1 \end{cases} \tag{20}$$

where Γ_i is the value of the ith category with probability $p(\Gamma_i)$ in the discrete-gamma distribution and its mean is equal to the average of $e^{w_{ab}}$ over all amino acid pairs and whose shape parameter is equal to α; $0 \leq \Gamma_i < \Gamma_{i+1}$. If $\Gamma_i < 1$ and $\gamma_i \exp w_{ab} \leq 1$ for $\forall a \neq b$, γ_i will be simply equal to a category in the discrete-gamma distribution whose mean is equal to 1.

Basically, categories with equal probability are also employed for the variation of selective constraint, but if $\Gamma_2/\langle\Gamma_i\rangle < 0.1$, two categories of Γ_1 and Γ_2 will be merged and replaced by their (weighted) mean category of $p(\Gamma_1) + p(\Gamma_2)$, and the largest value of Γ_i, Γ_m, will be divided into two categories of $p(\Gamma_m)/2$, virtually increasing the number of categories; that is, each category has unequal probability.

Datasets of protein sequences used to evaluate amino acid and codon substitution models

Substitution models are evaluated by employing three datasets of protein sequences; (1) fast-evolving interspecific mitochondrial proteins, (2) closely-related chloroplast-encoded proteins, and (3) fast-evolving HA proteins of Human Influenza A H1N1. These datasets are chosen, because the empirical amino acid substitution rate matrices, cpREV64 [14], mtREV [15], and FLU [16], that were designed as those specific to the respective protein sequences are available.

1 Dataset mammalian-mtProt: interspecific mammalian mitochondrial protein sequences concatenating 12 protein-coding genes from 69 mammalian species [17], whose genome sequences were obtained from the NCBI RefSeq database of organelle genomes. The alignment of each gene was made with the codon sequences by the modified version [2] of the ClustalW2 [18]. The total length of aligned genes is equal to 3618 amino acid sites, and the minimum amino acid identity between the sequences is equal to 0.66. The tree topology that was estimated as Tree-6 by [17] is used here as the most probable one. Overlapped segments between genes were removed from protein sequences.

2 Dataset cpProt-55: protein sequences concatenating 52 protein-coding genes from 55 chloroplast genomes of the major angiosperm lineages whose genome sequences are available in the NCBI RefSeq database out of the 64 genomes

analyzed in [19] and that are genes owned by all 55 taxa. The alignment of each gene was made with the codon sequences by the modified version [2] of the ClustalW2 [18]. The tree topology estimated by [19] is used as the most probable one in the present analysis. The total length of aligned genes is equal to 14128 amino acid sites, and the minimum amino acid identity between the sequences is equal to 0.73. The cpREV64 [14] was estimated from the full set of 77 protein-coding genes in the 64 genomes.

3 Dataset HA_Human-Flu-A-H1N1: Hemagglutinin proteins from the H1N1 type of human influenza A are downloaded from the entire influenza database at NCBI (July 2nd 2013 version). Only sequences with full length are retrieved and identical sequences are collapsed to single sequences; the number of the HA proteins was equal to 4231. These sequences were aligned by the MAFFT version 7 with the FFT-NS-2 option [20], and the tree topology assumed as the most probable one is the one inferred by the FastTree version 2 [21] with the JTT and CAT options. Also, virtually identical sequences were collapsed by removing sequences connected to the phylogenetic tree with branch length shorter than a certain threshold, 0.002. As a result, the number of the sequences was reduced to 1309. The multiple sequence alignment consists of 595 sites, of which 408 amino acid sites without gaps are used here, because sites with gaps were excluded in the estimation of FLU [16]. The FLU was estimated from the super set of influenza A proteins. ∼113000 influenza proteins consisting of ∼20 million residues.

Statistical comparison of amino acid and codon substitution models

Model selection must be pursued with considerable attention [22]. For the comparison of models one of which is a special case of the other, the likelihood ratio test (LRT) [23] can be used to test the superiority of a nesting model to nested models. Models that are not nesting or nested can be compared using Akaike information criterion (AIC) [24], Bayesian information criterion (BIC) [25], a decision-theoretical approach [26, 27], and Bayes factor [28]. Here, AIC and BIC for a given tree topology of aligned codon sequences are used to compare amino acid substitution models with empirical amino acid substitution rate matrices and the mechanistic codon substitution model with the wide range of selective constraint matrices. The AIC and BIC are defined as follows [29]:

$$\text{AIC} \equiv -2\ell(\hat{\boldsymbol{\theta}}) + 2K \quad (21)$$

$$\text{BIC} \equiv -2\ell(\hat{\boldsymbol{\theta}}) + K \log L \quad (22)$$

where K is the number of adjustable parameters, $\hat{\boldsymbol{\theta}}$ is the vector of the ML estimates of the parameters, $\ell(\hat{\boldsymbol{\theta}})$ is the maximum log-likelihood value, and L is the number of sites in an amino acid alignment. A model with a smaller value of AIC or BIC is considered to be a better model.

References

1. Miyazawa, S.: Selective constraints on amino acids estimated by a mechanistic codon substitution model with multiple nucleotide changes. PLoS One **6**, 17244 (2011)
2. Miyazawa, S.: Advantages of a mechanistic codon substitution model for evolutionary analysis of protein-coding sequences. PLoS One **6**, 28892 (2011)
3. Halpern, A.L., Bruno, W.J.: Evolutionary distances for protein-coding sequences: modeling site-specific residue frequencies. Mol. Biol. Evol. **15**, 910–917 (1998)
4. Yang, Z., Nielsen, R.: Mutation-selection models of codon substitution and their use to estimate selective strengths on codon usage. Mol. Biol. Evol. **25**, 568–579 (2008)
5. Miyata, T., Miyazawa, S., Yasunaga, T.: Two type of amino acid substitutions in protein evolution. J. Mol. Evol. **12**, 219–236 (1979)
6. Muse, S.V., Gaut, B.S.: Nucleotide substitution rates, with application to the chloroplast genome. Mol. Biol. Evol. **11**, 715–724 (1994)
7. Miyazawa, S., Jernigan, R.L.: A new substitution matrix for protein sequence searches based on contact frequencies in protein structures. Protein Eng. **6**, 267–278 (1993)
8. Goldman, N., Yang, Z.: A codon-based model of nucleotide substitution for protein-coding DNA. Mol. Biol. Evol. **11**, 725–736 (1994)
9. Yang, Z., Nielsen, R., Hasegawa, M.: Models of amino acid substitution and application to mitochondrial protein evolution. Mol. Biol. Evol. **15**, 1600–1611 (1998)
10. Whelan, S., Goldman, N.: Estimating the frequency of events that cause multiple-nucleotide changes. Genetics **167**, 2027–2043 (2004)
11. Yang, Z.: Maximum-likelihood estimation of phylogeny from DNA sequences when substitution rates differ over sites. Mol. Biol. Evol. **10**, 1396–1401 (1993)
12. Yang, Z.: Maximum likelihood phylogenetic estimation from DNA sequences with variable rates over sites: approximate methods. J. Mol. Evol. **39**, 306–314 (1994)
13. Yang, Z.: A space-time process model for the evolution of DNA sequences. Genetics **139**, 993–1005 (1995)
14. Zhong, B., Yonezawa, T., Zhong, Y., Hasegawa, M.: The position of gnetales among seed plants: overcoming pitfalls of chloroplast phylogenomics. Mol. Biol. Evol. **10**, 1093 (2010)
15. Adachi, J., Hasegawa, M.: Model of amino acid substitution in proteins encoded by mitochondrial DNA. J. Mol. Evol. **42**, 459–468 (1996)
16. Dang, C.C., Le, S.Q., Gascuel, O., Le, V.S.: Flu, an amino acid substitution model for influenza proteins. BMC Evol. Biol. **8**, 331 (2008)
17. Nikaido, M., Cao, Y., Harada, M., Okada, N., Hasegawa, M.: Mitochondrial phylogeny of hedgehogs and monophyly of eulipotyphla. Mol Phylogenet Evol **28**, 276–284 (2003)
18. Larkin, M.A., Blackshields, G., Brown, N.P., Chenna, R., McGettigan, P.A., McWilliam, H., Valentin, F., Wallace, I.M., Wilm, A., Lopez, R., Thompson, J.D., Gibson, T.J., Higgins, D.G.: Clustalw and clustalx version 2.0. Bioinformatics **23**, 2947–2948 (2007)
19. Jansen, R.K., Cai, Z., Raubeson, L.A., Daniell, H., dePamphilis, C.W., Leebens-Mack, J., Müller, K.F., Guisinger-Bellian, M., Haberle, R.C., Chumley, T.W., Lee, S.-B., Peery, R., McNeal, J.R., Kuehl, J.V., Boore, J.L.: Analysis of 81 genes from 64 plastid genomes resolves relationships in angiosperms and identifies genome-scale evolutionary patterns. Proc. Natl. Acad. Sci. USA **104**, 19369–19374 (2007)
20. Katoh, K., Standley, D.M.: Mafft multiple sequence alignment software version 7: Improvements in performance and usability. Mol. Biol. Evol. **30**, 772–780 (2013)
21. Price, M.N., Dehal, P.S., Arkin, A.P.: FastTree 2 - approximately maximum-likelihood trees for large alignments. PLoS One **5**, 9490 (2010)
22. Posada, D., Crandall, K.A.: Selecting the best-fit model of nucleotide substitution. Syst. Biol. **50**, 580–601 (2001)
23. Stuart, A., Ord, K.: Likelihood ratio tests and the general linear hypothesis. In: Kendall's Advanced Theory of Statistics vol. 2, 5th edn. Edward Arnold, London (1996)
24. Akaike, H.: A new look at the statistical model identification. IEEE Trans. Autom. Contr. **AC-19**, 716–723 (1974)
25. Schwarz, G.: Estimating the dimension of a model. Ann Stat **6**, 461–464 (1974)
26. Minin, V., Abdo, Z., Joyce, P., Sullivan, J.: Performance-based selection of likelihood models for phylogeny estimation. Syst. Biol. **52**, 674–683 (2003)
27. Abdo, Z., Minin, V.N., Joyce, P., Sullivan, J.: Accounting for uncertainty in the tree topology has little effect on the decision-theoretic approach to model selection in phylogeny estimation. Mol. Biol. Evol. **22**, 691–703 (2005)
28. Suchard, M.A., Weiss, R.E., Sinsheimer, J.S.: Bayesian selection of continuous-time Markov chain evolutionary models. Mol. Biol. Evol. **18**, 1001–1013 (2001)
29. Seo, T.K., Kishino, H.: Statistical comparison of nucleotide, amino acid, and codon substitution models for evolutionary analysis of protein-coding sequences. Syst. Biol. **58**, 199–210 (2009)
